# Clinical Trajectories of Suicide Attempts and Self-harm in Patients Admitted to Acute-care Hospitals in Japan: A Nationwide Inpatient Database Study

**DOI:** 10.2188/jea.JE20200018

**Published:** 2021-03-05

**Authors:** Hiroyuki Ohbe, Tadahiro Goto, Ryuichi Yamazaki, Taisuke Jo, Hiroki Matsui, Kiyohide Fushimi, Hideo Yasunaga

**Affiliations:** 1 Department of Clinical Epidemiology and Health Economics, School of Public Health, The University of Tokyo, Tokyo, Japan; 2 Graduate School of Medical Science, University of Fukui, Fukui, Japan; 3 Department of Psychiatry, The Jikei University School of Medicine, Tokyo, Japan; 4 Department of Health Services Research, Graduate School of Medicine, The University of Tokyo, Tokyo, Japan; 5 Department of Health Policy and Informatics, Tokyo Medical and Dental University Graduate School of Medicine, Tokyo, Japan

**Keywords:** suicide, suicide, attempted, suicide methods, Japan, acute care hospital

## Abstract

**Background:**

For patients with suicide attempts or self-harm, acute-care hospitals often function as the primary or sole point of contact with the healthcare system. However, little is known about patient characteristics or clinical trajectories of suicide attempts and self-harm episodes among those admitted to acute-care hospitals. This study aimed to describe the characteristics of suicide attempts and self-harm among patients admitted to acute-care hospitals, and the clinical practices provided in these hospitals, using a nationwide inpatient database in Japan.

**Methods:**

Using data from the Japanese Diagnosis Procedure Combination inpatient database from June 2015 to March 2017, we identified patients with emergency admission for suicide attempts or self-harm. We did not include patients with elective admission to psychiatric hospitals or outpatients. We described patient characteristics, treatments for physical injuries, psychiatric interventions, and discharge status.

**Results:**

We identified 17,881 eligible patients during the 22-month study period. Overall, 38% of the patients did not have any psychiatric or behavioral comorbidities at admission. The most common suicide method was drug overdose (50%), followed by hanging (18%), jumping from a height (13%), cutting or piercing without wrist cutting (7.1%), poisoning (6.6%), and wrist cutting (5.4%). Suicide was completed by 2,639 (15%) patients. Among patients discharged to home, 51% did not receive any psychiatric intervention. In 468 acute-care hospitals (54%), no psychiatric intervention was provided during the study period.

**Conclusion:**

We found that half of acute-care hospitals did not provide any hospital-based psychiatric care for patients with suicide attempts or self-harm.

## INTRODUCTION

Suicide is a major public health issue worldwide. In Japan, 21,321 people (16.8 per 100,000 persons) committed suicide in 2017,^1^ and Japan has the sixth highest suicide rate among Organization for Economic Co-operation and Development countries.^2^ A prior suicide attempt or self-harm episode is the single most important risk factor for completed suicide, and suicide risk has been reported to be 50 to 100 times higher in the year following a suicide attempt or instance of self-harm than the risk in the general population.^3^^,^^4^ The National General Principles of Suicide Prevention Policy in Japan identified the need for surveys on those who have attempted suicide or engaged in self-harm, as well as appropriate support for these individuals.^5^

One type of support for people with a history of suicide attempts or self-harm described in this policy is the enhancement of the psychiatric medical care system in emergency medical facilities.^5^ Because many suicide attempts and self-harm episodes result in emergency department visits, acute-care hospitals often function as the primary or sole point of contact with the healthcare system.^6^ Therefore, the Japan Cabinet Office has tried to establish a system of psychiatry liaison teams and coordination between general and psychiatric hospital departments through the medical fee revision of 2012.^5^ However, little is known about the patient characteristics or the current status of clinical management for these patients in Japan.

Previous descriptive studies on suicide attempts and self-harm have been conducted only in single institutions.^7^^–^^11^ A systematic review of descriptive studies of suicide attempters in emergency departments in Japan described these patients’ characteristics; however, the results of this study may have been biased because the review included only published articles.^12^ No previous study has examined the psychiatric medical care system after individuals who have attempted suicide or engaged in self-harm are admitted to acute-care hospitals. Therefore, this study aimed to describe the characteristics and clinical trajectories of suicide attempts and self-harm episodes using a nationally representative sample of patients admitted to acute-care hospitals in a nationwide inpatient database in Japan.

## METHODS

This study was a retrospective nationwide cohort study in Japan. The reporting of this study conforms to the RECORD statement. The Institutional Review Board of the University of Tokyo approved the study (approval number: 3501-[3] [December 25th, 2017]). No information allowing the identification of individual patients, hospitals, or physicians was obtained. The requirement for informed consent was waived because of the anonymous nature of the data.

### Data source

We used the Japanese Diagnosis Procedure Combination inpatient database, which includes discharge abstracts and administrative claims data. For 2016, this database contains information from 1,200 acute-care hospitals, covering 90% of all beds for critically ill patients and 70% of all beds for acute-phase patients throughout Japan. This database does not include patients admitted to psychiatric hospitals without need for general ward admission to treat physical injuries. The database includes information on age, sex, diagnoses (including main diagnosis, comorbidities present at admission, and conditions arising after admission), procedures, and discharge status. In addition, from June 2015 to March 2017, it was mandatory for health care providers to input codes for suicide attempts and self-harm for all patients admitted to acute-care hospitals, regardless of whether or not the admission was specifically for suicide attempt or self-harm. In this code, the definition of suicide attempt/self-harm specifies an intent to die or to engage in self-directed, potentially injurious behavior. Suicide methods were categorized as (i) hanging; (ii) jumping from a height; (iii) poisoning; (iv) drug overdose; (v) cutting or piercing without wrist cutting; (vi) wrist cutting; (vii) other; and (viii) unspecified. When health care providers could not confirm at admission that the behavior was a suicide attempt or self-harm, they assigned the code of (ix) none.

In the Japanese Diagnosis Procedure Combination inpatient database, diagnoses are recorded using International Classification of Diseases Tenth Revision (ICD-10) codes and written in Japanese text. We also extracted data from the *Annual Report for Functions of Medical Institutions 2016*, which contains all facility information and statistics.^13^

### Study population

We identified patients hospitalized for suicide attempts or self-harm from June 2015 to March 2017 using the codes for suicide or suicide attempt in the database (ie, we did not use ICD-10 codes to identify suicide attempts). Again, the database does not include outpatients or patients who were admitted only to a psychiatric hospital. We excluded (i) patients with unknown suicide methods; (ii) those aged <10 years, following previous literature^14^; (iii) patients whose data could not be combined with the *Annual Report for Functions of Medical Institutions 2016*; and (iv) patients who were admitted to primary emergency facilities. To account for history of suicide attempts, when patients were admitted for suicide attempt or self-harm multiple times during the study period, we used the last admission.

### Variables

Data on patient characteristics included age, sex, pregnancy, Japan Coma Scale (JCS) at admission,^15^ Charlson comorbidity index at admission (0, 1, 2, or ≥3),^16^ comorbid mental or behavioral disorders at admission, past history of suicide attempts, prior hospital admission for any reason within 1 year, suicide method, ambulance use, academic hospital, and type of emergency facilities. Comorbid mental or behavioral disorders at admission were identified using ICD-10 code-based diagnoses. Prior suicide attempts were identified from either prior admission with the codes for suicide/self-harm or the ICD-10 codes for comorbidities in suicide cases, following previous studies.^17^^,^^18^

Clinical practices, psychiatric interventions, and hospital characteristics were also identified. Psychiatric interventions during hospitalization included psychiatric ward admission, psychotherapy with a psychiatrist, consultation-liaison psychiatry, and the use of antidepressants. Hospital-based psychiatric interventions were defined as psychiatric interventions performed on eligible patients in a hospital at least once during the study period.

### Statistical analyses

We present patient characteristics, clinical practices, psychiatric interventions, and hospital characteristics by suicide method because lethality of suicide method is strongly correlated with patient characteristics, including impulsivity, intentness, and psychiatric severity.^19^^–^^21^ Suicide methods were classified as high lethality (hanging or jumping from a height) and low lethality (poisoning, drug overdose, cutting or piercing without wrist cutting, or wrist cutting).^22^^,^^23^ Psychiatric interventions during hospitalization are presented only for patients discharged to home. We also present the hospital characteristics by type of emergency facilities (secondary or tertiary).

Values of categorical variables are reported as counts (%), and values of continuous variables are reported as medians with interquartile range (IQR). All analyses were performed using Stata/MP 15.0 (StataCorp, College Station, TX, USA).

## RESULTS

**Figure 1.**
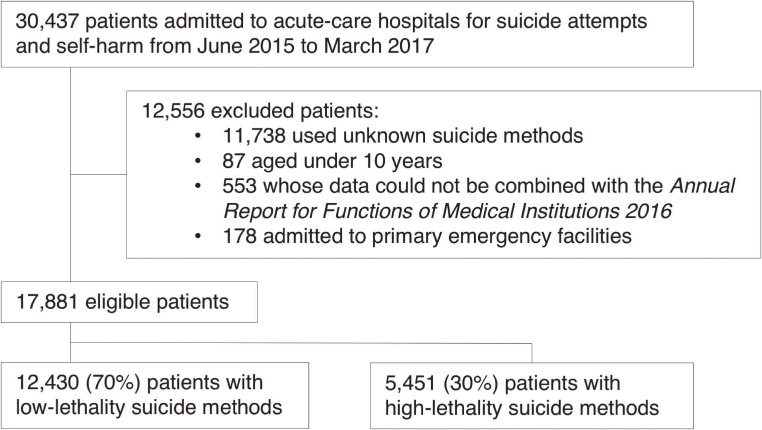
Patient flowchart

A total of 17,881 patients met our eligibility criteria during the 22-month study period (Figure 1).

Table 1 and Table 2 show patient characteristics and management. The study population had a median age of 43 years (IQR, 29–60), with men and boys making up 39% of the sample. Overall, 83% of the patients did not have any physical comorbidities at admission, and 38% of the patients did not have any psychiatric or behavioral comorbidities at admission. The most common comorbid mental or behavioral disorder at admission was depressive disorder. The most common suicide method was drug overdose (50%), followed by hanging (18%), jumping from a height (13%), cutting or piercing without wrist cutting (7.1%), poisoning (6.6%), and wrist cutting (5.4%). Suicide was completed by 2,639 patients (15%). Among patients discharged to home, 6,308 (51%) did not receive any psychiatric interventions during their hospitalization (Table 3).

**Table 1.** Patient characteristics

	Overall	Low-lethality suicide methods	High-lethality suicide methods
	(*n* = 17,881)	(*n* = 12,430)	(*n* = 5,451)
Age, years, median (IQR)	43 (29–60)	41 (28–56)	49 (31–68)
Male sex	6,891 (39%)	4,097 (33%)	2,794 (51%)
Pregnancy	140 (0.8%)	91 (0.7%)	49 (0.9%)
Japan Coma Scale at admission			
Alert consciousness	5,919 (33%)	3,906 (31%)	2,013 (37%)
Dizziness	3,868 (22%)	3,170 (26%)	698 (13%)
Somnolence	2,677 (15%)	2,418 (20%)	259 (4.8%)
Coma	5,417 (30%)	2,936 (24%)	2,481 (46%)
Charlson comorbidity index			
0	14,801 (83%)	10,319 (83%)	4,482 (82%)
1	2,055 (11%)	1,464 (12%)	591 (11%)
2	641 (3.6%)	420 (3.4%)	221 (4.1%)
≥3	384 (2.2%)	227 (1.8%)	157 (2.9%)
Comorbid psychiatric disorders			
Substance use	801 (4.5%)	733 (5.9%)	68 (1.2%)
Schizophrenia	2,592 (15%)	1,952 (16%)	640 (12%)
Bipolar affective disorder	1,055 (5.9%)	918 (7.4%)	137 (2.5%)
Depressive disorder	5,107 (29%)	4,078 (33%)	1,029 (19%)
Other mood disorder	263 (1.5%)	222 (1.8%)	41 (0.8%)
Stress-related disorder	2,443 (14%)	2,016 (16%)	427 (7.8%)
Personality disorder	459 (2.6%)	2,026 (16%)	430 (7.8%)
Other psychiatric disorder	1,272 (7.1%)	1,389 (11%)	314 (5.8%)
None	6,725 (38%)	3,500 (28%)	3,225 (59%)
Prior suicide attempts	980 (5.5%)	744 (6.0%)	236 (4.3%)
Prior admission within 1 year			
1–30 days	2,003 (11%)	1,431 (12%)	572 (11%)
31–365 days	1,866 (10%)	1,386 (11%)	480 (8.8%)
Suicide methods			
Hanging	3,184 (18%)	—	3,184 (58%)
Jumping from a height	2,267 (13%)	—	2,267 (42%)
Poisoning	1,188 (6.6%)	1,188 (9.6%)	—
Drug overdose	9,007 (50%)	9,007 (73%)	—
Cutting or piercing without wrist cutting	1,269 (7.1%)	1,269 (10%)	—
Wrist cutting	966 (5.4%)	966 (7.8%)	—
Ambulance use	14,001 (78%)	9,921 (80%)	4,080 (75%)
Academic hospital	4,634 (26%)	3,077 (25%)	1,557 (29%)
Type of emergency facilities			
Tertiary	12,125 (68%)	8,265 (67%)	3,860 (71%)
Secondary	5,756 (32%)	4,165 (34%)	1,591 (29%)

IQR, interquartile range.

**Table 2.** Details of clinical practice and discharge status

	Overall	Low-lethality suicide methods	High-lethality suicide methods
	(*n* = 17,881)	(*n* = 12,430)	(*n* = 5,451)
Admission to psychiatric ward	3,534 (20%)	2,652 (21%)	882 (16%)
Forced hospitalization by licensed psychiatrist	1,486 (8.3%)	1,059 (8.5%)	427 (7.8%)
Medical department			
Internal medicine	5,061 (28%)	3,901 (31%)	1,160 (21%)
Surgery	2,636 (15%)	1,242 (10%)	1,394 (26%)
Emergency medicine	6,623 (37%)	4,701 (38%)	1,922 (35%)
Psychiatry	2,657 (15%)	2,004 (16%)	653 (12%)
Other	904 (5.0%)	582 (4.7%)	322 (5.9%)
Intensive care unit admission	3,919 (22%)	2,420 (20%)	1,499 (28%)
Use of vasopressors	2,891 (16%)	691 (5.6%)	2,200 (40%)
Mechanical ventilation	3,510 (20%)	1,454 (12%)	2,056 (38%)
Renal replacement therapy	318 (1.8%)	277 (2.2%)	41 (0.8%)
Blood transfusion	1,079 (6.0%)	463 (3.7%)	616 (11%)
Discharge location			
Discharge to home	12,368 (69%)	10,242 (82%)	2,126 (39%)
Transfer to a different hospital or nursing home	2,874 (16%)	1,854 (15%)	1,020 (19%)
In-hospital death	2,639 (15%)	334 (2.7%)	2,305 (42%)
Death within 24 hours	2,234 (12%)	188 (1.5%)	2,046 (38%)
Length of stay (in days), median (IQR)	3 (2–11)	3 (2–8)	4 (1–23)
Total health care cost (in USD), median (IQR)	2,706 (1,508–6,573)	2,587 (1,505–5,420)	3,359 (1,514–12,416)

IQR, interquartile range; USD, United States dollars.

**Table 3.** Psychiatric interventions during hospitalization among patients discharged to home

	Overall	Low-lethality suicide methods	High-lethality suicide methods
	(*n* = 12,368)	(*n* = 10,242)	(*n* = 2,126)
Psychiatric ward admission	2,910 (24%)	2,273 (22%)	637 (30%)
Psychotherapy with psychiatrist	5,372 (43%)	4,447 (43%)	925 (44%)
Consultation-liaison psychiatry	305 (2.5%)	234 (2.3%)	71 (3.3%)
Antidepressant use	2,314 (19%)	1,771 (17%)	543 (26%)
None of these interventions	6,308 (51%)	5,241 (51%)	1,067 (50%)

We categorized patients into a low-lethality suicide methods group (*n* = 12,430, 70%) and a high-lethality suicide methods group (*n* = 5,451, 30%). In the low-lethality suicide methods group, patients were relatively young and more likely to be female (67%). Most of these patients had a comorbid mental or behavioral disorder at admission (72%), with depressive disorder (33%) being especially common. In-hospital mortality was low, and length of stay was short. Most of these patients were discharged to home, and half received psychotherapy during hospitalization.

In the high-lethality suicide methods group, patients tended to be middle-aged. Fewer than half of these patients had comorbid mental or behavioral disorders at admission. They often required intensive care unit admission and invasive interventions. This group had high in-hospital mortality (42%), especially within 24 hours of admission (38%). One-third of survivors were transferred to a different hospital. About half of the patients discharged to home did not receive psychotherapy during hospitalization.

Table 4 shows the characteristics of hospitals where acute-care hospitalization for suicide attempts or self-harm was provided. The median number of patients admitted for suicide attempts or self-harm during the study period was 10 (IQR, 4–33). In 468 hospitals (54%), no psychiatric intervention was provided during the study period. Secondary emergency facilities were less likely to provide psychiatric intervention during hospitalization than were tertiary emergency facilities.

**Table 4.** Characteristics of hospitals that provided acute-care hospitalization for suicide attempt and self-harm

	Overall	Tertiary emergency facilities	Secondary emergency facilities
	(*n* = 868)	(*n* = 224)	(*n* = 644)
Numbers of hospital beds, median (IQR)	326 (201–500)	577 (456–700)	275 (188–378)
Annual number of ambulance transports, median (IQR)	2,394 (1,329–4,146)	4,452 (2,894–6,128)	1,953 (1,063–3,165)
Academic hospital	76 (8.8%)	54 (24%)	22 (3.4%)
Number of patients admitted for suicide attempt/self-harm during the study period, median (IQR)	10 (4, 33)	39 (23, 75)	4 (2, 10)
Hospital-based psychiatric interventions			
Forced hospitalization by licensed psychiatrist	204 (24%)	109 (49%)	95 (15%)
Psychotherapy with psychiatrist	371 (43%)	189 (84%)	182 (28%)
Consultation-liaison psychiatry	59 (6.8%)	44 (20%)	15 (2.3%)
None of these interventions	468 (54%)	30 (13%)	438 (68%)

IQR, interquartile range; USD, United States dollars.

## DISCUSSION

Using a nationwide database in Japan, we described the overall patient characteristics and the management of patients admitted to acute-care hospitals for suicide attempts or self-harm in a large Japanese population. To the best of our knowledge, this is the first study of its kind. Our results have several implications for current practices.

First, our study revealed that 54% of acute-care hospitals did not have any hospital-based psychiatric care system in place, despite admitting patients who were at high risk for attempting suicide or engaging in self-harm. Easy access to health care—especially psychiatric care—is an established protective factor against suicide.^24^ According to previous studies, psychiatric interventions following self-harm may reduce subsequent repetition of self-harm.^25^^,^^26^ During hospitalization, a psychiatric care system in acute-care hospitals can provide prompt medical/social crisis intervention, grasp emergency response needs, and ensure timely service provision by, for example, local social resources. Even when hospitals without a psychiatric care system refer suicide attempters to hospitals with psychiatric departments after the physical injury is treated, it is challenging to determine whether these patients need emergency psychiatric hospitalization. Furthermore, failure to adhere to discharge recommendations is common among patients who have attempted suicide.^27^ Although psychiatric care systems are not always available and are costly, our findings suggest that it may be necessary to continue promoting access to psychiatric care systems and evaluating the psychiatric care in acute-care hospitals using nationwide data.

Second, we found that, in the low-lethality suicide methods group, some patients (such as those with wrist cutting or drug overdose) may not have needed hospitalization to treat physical injuries. The World Health Organization has recommended against routine hospitalization in non-specialized departments of general hospitals for persons who have engaged in self-harm but who do not need physical treatment, with the goal of preventing further acts of self-harm.^28^ Hospitalization is a costly treatment option, both financially and otherwise; therefore, more education about suicide attempts and self-harm is encouraged for non-psychiatric physicians.^29^

Third, our study showed that 50% of patients discharged to home in the high-lethality suicide methods group did not receive any psychiatric interventions during their hospitalization. However, a previous national cohort study revealed that patients with a prior high-lethality suicide attempt were three to six times more likely to complete suicide, compared with those with a prior low-lethality suicide attempt.^14^ Intensive aftercare is, therefore, warranted following suicide attempts, especially among surviving patients who have used high-lethality methods.

This study had several limitations. First, our study did not include patients who had attempted suicide or engaged in self-harm who were treated in emergency departments without being hospitalized or those who were hospitalized only in psychiatric hospitals. Thus, our results may present a distorted view of the epidemiology of suicide attempts and self-harm. Second, the identification of suicide attempts and self-harm in our study did not follow the internationally accepted definition of “deliberate self-harm”^30^ because of the lack of rigorous terminology and definitions in our database. Third, we did not account for repeated suicide attempts/self-harm or suicide mortality over a long period because of the short study duration.

In conclusion, using a nationwide database in Japan, we described the patient characteristics and clinical trajectories of suicide attempts and self-harm among individuals admitted to acute-care hospitals in a large Japanese population. We found that half of acute-care hospitals did not have any hospital-based psychiatric care system to treat patients who had attempted suicide or engaged in self-harm.
